# Psychometrics for physicians: everything a clinician needs to know about assessments in medical education

**DOI:** 10.5116/ijme.625f.bfb1

**Published:** 2022-04-22

**Authors:** Mohsen Tavakol, David O'Brien

**Affiliations:** 1 Medical Education Centre, School of Medicine, The University of Nottingham, UK

**Keywords:** Psychometrics, assessment, medical education

## Abstract

Assessments in medical education, with consequent decisions about performance and competence, have both a profound and far-reaching impact on students and their future careers. Physicians who make decisions about students must be confident that these decisions are based on objective, valid and reliable evidence and are thus fair. An increasing use of psychometrics has aimed to minimise measurement bias as a major threat to fairness in testing. Currently, there is substantial literature on psychometric methods and their applications, ranging from basic to advanced, outlining how assessment providers can improve their exams to make them fairer and minimise the errors attached to assessments. Understanding the mathematical models of some of these methods may be difficult for some assessment providers, and in particular clinicians. This guide requires no prior knowledge of mathematics and describes some of the key methods used to improve and develop assessments; essential for those involved in interpreting assessment results. This article aligns each method to the Standards for educational and psychological testing framework, recognised as the gold standard for testing guidance since the 1960s. This helps the reader develop a deeper understanding of how assessors provide evidence for reliability and validity with consideration to test construction, evaluation, fairness, application, and consequences, and provides a platform to better understand the literature in regards other more complex psychometric concepts that are not specifically covered in this article.

## Introduction

As competency-based assessments grow in importance, particularly amongst professional groups and those responsible for licencing and regulation of individuals, various standards for educators developing such assessments have been created to guide improvement. For example, the Standards for Educational and Psychological Testing, referred to hereafter as the Standards, provide a gold standard aiming to "promote sound testing practices and to provide a basis for evaluating the quality of those practices".^1^  However, the assessment capabilities of many teachers often do not fit the standards adopted by such professional bodies.^2^ Physicians involved in teaching and assessments need to understand and use these critical Standards in improving fairness in assessments and confirming professional competence. Many physicians will have substantial experience in contributing to applied knowledge tests and OSCEs in both undergraduate and postgraduate medical education settings. Some may be directly involved in designing assessment questions, OSCE stations or identifying pass marks using various approaches, but few probably ever ask themselves, does this assessment truly measure what it was intended to measure? This question is fundamental in providing evidence of validity and reliability of any assessment and improving undergraduate and postgraduate medical education curricula. In fact, the General Medical Council UK states that, "In developing and reviewing assessment methods, medical schools should consider the validity, reliability/generalisability, feasibility, fairness, educational impact, cost-effectiveness, acceptability and defensibility" of assessments,^3^ essentially mandating that assessment providers must offer validity and reliability evidence for their assessments. Moreover, decisions made about students' marks have a significant impact on their future careers, so in order to be confident that any decisions are fair, assessment providers must first ensure that assessments on which these decisions are based are again both valid and reliable.

"Assessment is the systematic process of collecting and interpreting information to make decisions."^2^ High-quality assessments provide valid and reliable evidence about student performance and assist medical educators in determining the effectiveness of their instructional strategies.

Determining the quality of assessments and providing objective evidence of reliability for any assessment requires post-examination analysis of exam data after assessments have been undertaken by students and is a means for improving the exam cycle.^4^ This includes not only providing validity and reliability evidence for test scores, but also detecting ambiguous assessment questions, minimising sources of error and developing fair assessment questions.^5^^-^^8^ Variations in fairness in measurement quality is assessed in the Standards by considering; "fairness as the lack or absence of measurement bias, fairness as access to the constructs measured, fairness as validity of individual test score interpretations for the intended use(s), and  fairness as the equality of testing outcomes for relevant test taker subgroups."^1^

This paper aims to provide a guide for physicians as assessment providers to help them better understand how to deliver high-quality assessments and minimise construct-irrelevant variance. Construct-irrelevant variance refers to any irrelevant or uncontrolled factors in assessments, such as poorly designed questions or a student randomly answering a question correctly, resulting in an increase or decrease in marks for certain students which may lead to misinterpretation of the test results.  This is obviously undesirable. Standard 3.0 states, "all steps in the testing process, including test design, validation, development, administration, and scoring procedures, should be designed in such a manner as to minimize construct-irrelevant variance and to promote valid score interpretations for the intended use for all examinees in the intended population".^1^ 

This article also helps physicians interpret psychometric reports provided by their own medical schools, when sitting on moderation committees or working as external examiners in ensuring the quality of assessments at other medical schools.

### Descriptive statistics of student marks

Standard 1.18 asserts that "when a certain level of test performance predicts adequate or inadequate criterion performance, information about the levels of criterion performance associated with given levels of test scores should be provided."^1^ It stands to reason therefore, that after administrating any test, the first step is to apply descriptive statistics to describe the exam data. Students' marks are meaningless in isolation unless we first condense them into a more understandable style by using statistics to summarise the exam data and marks. Such descriptive statistics include the mean, standard deviation, minimum mark and maximum mark, and provide an overall understanding of the student marks and, importantly, their distribution around the mean.

For example, calculating the mean or average mark can be used to compare the position of an individual student to the mean. For instance, if Rose received a mark of 85 out of 100 and the average or mean mark is 80, we could conclude she scored five marks above the average mark (i.e., 85-80=5). Another use of the achieved mean mark is to compare it to the pass mark set for the exam. If the pass mark is significantly greater than the mean mark, the exam may be difficult for all students. If this is the case, we may get an exceptional failure rate. A major limitation of the mean is that it is sensitive to all marks. In situations where we have outliers and extreme marks, we may therefore get a misleading figure of the distribution of marks and in such cases the median is often the best statistic to use, as it is not sensitive to outliers.

We could also calculate the minimum (smallest value) mark and maximum (largest value) mark of all students' marks. Based on these values, we can immediately see the weakest and strongest students in the cohort of interest. Furthermore, suppose the maximum mark obtained was significantly lower than the total mark available in a test, this may indicate that the test was too difficult, or that some learning objectives might not have been taught, and therefore some questions were beyond the students' ability. Consequently, a review of the difficulty of the assessment is also required and will be discussed in the following sections.

**Figure 1:**
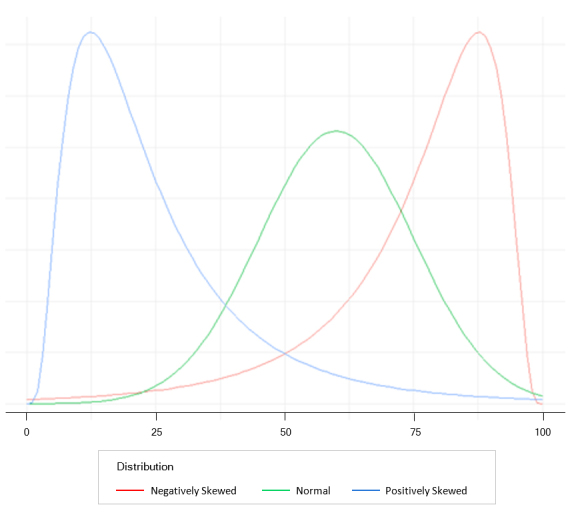
Mark distribution patterns using histograms

One statistic used to understand the distribution of marks is the standard deviation (SD) and tells us how variable or spread out the marks are. An SD of zero tells us all marks are the same, a small SD tells us all marks are close to the mean, and a large SD tells us that marks are distinct from the mean. Practically, SD is very useful when the sample size is large, and marks are normally distributed in a bell-shaped curve, as indicated in the green graph in Figure 1. In a normally distributed sample, the mean, median and mode are all equal values, and approximately 68% of the marks lie between ±1SD, approximately 95% of the marks are between ±2SD, and about 99% of the marks lie within ±3SD. Consider if the mean of a test is 50 and the SD is 10. A student receives a mark of 65. If the test is normally distributed, then the location of the student based on a bell-shaped curve is above +1SD from the mean (50+10=60), which is "good"!

If students' marks are normally distributed, then the classification of student performance is straightforward. For example, those who received a mark less than -3SD are considered low performers. Those receiving a mark between ±1SD may be categorised as average students, and those who received a mark greater than +1SD may be classified as high performers.

The use of a histogram as a graphical representation of the frequency of marks by using bars of different heights allows the distribution of students' marks to be easily examined visually. For example, if the distribution of marks is positively skewed (Figure 1 blue curve), then most marks are at the low end of the distribution, and the test was too difficult. Therefore, we expect to see the mean mark greater than the median mark. Hence, we may need to review the teaching process or those setting the assessment as it seems that something went wrong. However, if the distribution of marks is negatively skewed (Figure 1 red curve), then most marks are at the high end of the distribution, and the test was too easy with the students achieving all learning objectives of the test. Therefore, we expect to see the mean mark lower than the median mark. When student marks are strictly skewed, the mean may provide a misleading figure of the marks and the median is therefore a more useful measure.

Whilst descriptive statistics show us measures of central tendency, e.g., mean and median and measures of variability (the spread of marks), e.g., SD, they do not tell us how accurate the marks are? This question is a matter of reliability of test marks obtained from assessments and is discussed below.

### Providing reliability evidence

The validity of an assessment is the ability of a test to measure what it claims to measure. If a test is not valid, then it cannot be reliable, whereas a test can be reliable, in that the results are consistent and reproducible without the test being valid!  The Standards place great importance on the reliability of test scores, clearly stating that "appropriate evidence of reliability/precision should be provided for the interpretation for each intended score use".^1^ Therefore, providing reliability evidence is an integral part of reviewing any assessment. Test-score reliability provides valuable information concerning the trustworthiness of student marks. If assessment providers want to ensure their assessments are fair and reliable, they need to provide their assessments' reliability and validity evidence.

Importantly, there is an association between future student performance and test-score reliability. Assessment providers should be accountable to students and external agencies, and if challenged, provide defensible and robust evidence regarding the test-score reliability of their assessments. Many students will have graduated with substantial debt and have an expectation that assessment providers can defend the assessments they have designed, be that OSCEs or applied knowledge tests.^2^ In order to design reliable assessments, physicians are required to have a deeper understanding of the concept of 'test-score reliability'.

If a test is consistent and stable, it is also predictable and accurate, and so is said to be reliable. As noted previously, test-score reliability refers to the ability of an assessment to measure a concept consistently. It is noteworthy that reliability relates to the assessment results, not the assessment tool itself. Reliability is thus concerned with the consistency of the test results.^9^ To put it simply, if assessors independently rate students on the same assessment task and obtain similar ratings, we could conclude that our assessment results have a high degree of reliability from one assessor to another. Unfortunately, no assessment can be 100% reliable, as no assessment or measurement is perfect or free from error. Understandably, the amount of error shrinks the usefulness of an assessment, and therefore, although we cannot eliminate measurement error from our assessment entirely, we can and should pinpoint, isolate, and estimate the source of errors. The goal of test construction is to minimise any measurement error and therefore increase test-score reliability. As the measurement error reduces, confidence about the trustworthiness and reliability of assessment results increases. Errors of measurement which can confound students' marks can be split into two groups: Internal and external errors. There are many sources of internal and external errors. Internal errors could be, for example, poorly worded or ambiguous questions or poor OSCE station design. External errors are external to the test itself, for example, room temperature and lack of sleep for students during assessment periods.

Having stressed that although we cannot wholly remove measurement error from our assessments, we should aim to estimate how much error is present in any specific test. Developed in 1951 to measure the internal consistency of a test or a scale,^10^ Cronbach's alpha is widely used in medical education to measure the reliability of test scores. Cronbach's alpha is sometimes called coefficient alpha or just alpha, and a detailed description is beyond the scope of this paper. Readers who are interested in the details of Cronbach's alpha may refer to these articles.^11^^,^^12^

Cronbach's alpha is usually supplied to assessment providers by psychometricians and ranges between 0 and 1. A zero value suggests a lack of test-score reliability, i.e., exam data are just random data and useless. Values closer to 1 tell us the assessment has a good test-score reliability and can therefore be used to discriminate between high and low performers. An acceptable alpha is between 0.70 and 0.90. The higher the alpha, the higher the reliability of test scores. It should be noted, however, that a very high alpha, e.g., 0.98, does not always mean a high degree of internal consistency and may just indicate question redundancy, i.e., questions assessing the same topic or repeating information provided by other questions in the test which we may wish to remove.

Physicians should therefore know the following about test score reliability:

1) Any item in a particular assessment can be a source of error, and errors lead to the unreliability of test scores.

2) The reliability of test scores is a function of the test length. As the number of assessment questions increases, the error will usually decrease, and so the reliability of test scores should thus increase. If your test is too short, you may get a low alpha and low reliability. If you wish to increase the alpha, increase the number of assessment questions. There is, however, a balance to be struck here. Tests that are too long can result in student fatigue, when paradoxically reliability may even go down, which is obviously undesirable. Modelling the number of questions required based on the learning outcomes within the allotted time is a skill of good assessment design.

The quality of assessment questions is a major factor that can also affect the reliability of test scores. Item analysis can be used to measure the quality of assessment questions. To put it simply, item analysis improves the quality of an assessment by uncovering vague, ambiguous, tricky, too easy or too difficult questions. Item analysis tells us which questions may be too easy or too difficult (beyond the student ability) and which questions do not discriminate between high and low performers. A test based on item analysis has more reliability than a test whose questions are not based on item analysis. So, the aim of item analysis is to detect problematic questions within a test. 'Problematic questions' are sometimes referred to as 'bad', 'faulty', 'flawed', 'misfitting' or 'underperforming questions' and often have been developed poorly, causing students confusion when responding to them. Item analysis provides quantitative and objective evidence about assessment questions, i.e., which question should be adopted, and which question should be revised or discarded. Item analysis, i.e., item difficulty and item discrimination, is discussed in more detail later.

### Standard Error of Measurement

As discussed above, reliability is the degree to which a score is consistent across assessors, situations, or assessment forms. For example, when different assessors rate students on an OSCE station with the same learning objectives, assessors should not be overly harsh ("hawkish") or lenient ("dovish") as student performance should not depend on the assessor, situation, assessment form or other external factors but on their own ability. Such inconsistent judgment and accuracy on the part of the assessor causes the student's observed score to deviate from their true score. This results in an increase in the false-positive rate (those who passed the test but should not have passed the test) or the false-negative rate (those who failed the test but should not have failed the test). This disparity between the true and observed scores is called the Standard Error of Measurement (SEM). The Standards state that, "When considering the reliability/precision of test scores for relevant subgroups, it is useful to evaluate and report the standard error of measurement as well as any coefficients that are estimated."^1^

Suppose a student is tested ten times (without memory, exercise effects, or other change). If an average of the measurements is taken, a reliable estimate of the student's true score will be obtained. The average score observed will be approximately his or her true score, and the scores observed will be distributed almost normally around the true score. However, it is impossible to ask students to take an exam multiple times to estimate their true scores. The SEM is applied to student scores to establish confidence bands for estimating their true score. For example, suppose a student receives a score of 80 in a cardiology exam. The standard deviation of the errors of measurement, i.e., SEM for the exam, is calculated as 4. Now, you are in a position to establish the confidence band for the student, which is 80 ± 1 SEM= 76 to 84. Considering the empirical rule with a normal distribution, approximately 68% of the observed scores are within one SEM of the student's true score; approximately 95% are within two SEM, and about 99.7% are within three SEM. It is critical to note that the smaller the SEM, the narrower the confidence band, indicating a high reliability of the test scores and assurance that students' observed scores are close to their true scores.^9^

Although the SEM is widely used in medical education assessment, it has its limitations. Therefore, the SEM is smaller for students in whom the level of difficulty of the assessment questions is appropriate, and SEM does not work very well for extreme scores (high or low). For example, if an MCQ test is very challenging for a student, the student score is very likely to be based on their luck in guessing the correct answers rather than their ability.

### Adjustment to pass mark

Such is the importance of the pass mark as a significant boundary between passing and failing or in identifying proficiency skill levels, that this is addressed by several standards in the Standards document. As noted previously, in order to make recommendations about pass marks, Standard 5.0 states that "test scores should be derived in a way that supports the interpretations of test scores for the proposed uses of tests. Test developers and users should document evidence of fairness, reliability, and validity of test scores for their proposed use."^1^

An important point raised in the Standards is that when the pass mark is to be based on the judgment of examiners, for instance during an OSCE, that the qualification of standard setters and the standard-setting method used must be documented. Standard setters should understand "what they are to do and that their judgments are as thoughtful and objective as possible".^1^ Although standard setters should be well-versed and have a deep understanding of standard-setting methods in order to judge exam questions well, all subjective interpretation is inevitably prone to error, with inconsistency across the ratings of standard setters resulting in an overestimation or underestimation of the pass mark. It may not accurately classify students, resulting in false positive and false negative decisions followed by adverse consequences.^13^^, ^^14^ The pass mark calculated by standard setters is therefore not necessary the one ultimately used and may require adjustment to take account of any errors in order to make it fair for all students.

Adjustment can be made in a number of ways, but for knowledge examinations is most commonly achieved by considering the SEM. The SEM is calculated by subtracting 1 from the reliability of a test score, taking the square root of this and then multiplying it by the standard deviation of the test. SEM calculation should be performed after the test is administered. The commonest way of adjusting a pass mark using the SEM is to apply SEM to the pass mark calculated for the test, by either increasing or decreasing the pass mark by a multiple of the SEM for the exam (e.g. +/- 1, 2 or 3 SEM as noted previously),^14^^,^^15^ and sometimes referred to as "giving the examinee the benefit of the doubt."^15^ Using the cardiology test noted previously as an example, with a calculated pass mark of 80 and a SEM of 4, if the test was considered too difficult and too few students passed, the pass mark could be adjusted down by 1 SEM to 76, or by 2 SEM to 72. A note of caution here is if the SEM is high. This suggests a lot of ‘noise’ has been introduced into the mark and there may be issues with internal consistency and reliability which is not desirable, in which case using such a high SEM would not be advisable to adjust the pass mark.

A distinction must also be made between high performers whose low marks do not reflect their actual performance and low performers whose high marks do not reflect their actual performance. Therefore, to avoid any negative consequences of the adjustments on false positive and false negative decisions, the decision to apply one, two or three SEMs below the Angoff average should be based on various statistical and subjective judgments, e.g., the size of the SEM and the internal consistency of the standard setters and the subjective judgement of expert examiners during discussion during an examination moderation committee for example. It is critical to provide validity evidence based on the intended and unintended outcomes of the distribution of marks and assessment results before and after the adjustments.^14^ This may need to take into account issues such as patient safety, policy documentation, public and student satisfaction for example when deciding to change the pass mark and any impact should be discussed to reach subjective agreement by the moderation group responsible for overseeing that specific examination.

Although the SEM is widely used in medical education, another option for adjustment is to use the standard error of judgment, when the pass marks are identified independently by standard setters. This may estimate more errors than other methods, related to these subjective judgments, especially if additional feedback is given to the standard setters. Sometimes, assessment providers may look at the range of the pass marks provided by standard setters for any possible outliers and choose a pass mark within the interquartile range of the pass marks estimated by individual standard setters.

In Objective Structured Clinical Examinations (OSCEs), rather than using SEM or standard error of judgement, the Standard Error of the Regression (SER or S),  also known as the Standard Error of the Estimate, is frequently used to adjust the pass mark when a borderline regression approach is used.^16^  The SER in essence a measure of the precision of predictions from the observed marks and the model used, so in the borderline regression model is then essentially the average distance at which the observed values deviate from the regression line. In this approach, the SER is used to adjust the pass mark if the evidence shows an examiner bias effect at a particular station, i.e., either a 'hawkish' or 'dovish' examiner.

With any method of adjustment, we must keep in mind that all pass marks are arbitrary and the product of judgments to some degree. In any high-stakes medical examination where the impact of passing of failing is significant for both the individual and the general public, provided that the determination of an adjusted pass mark is unbiassed and not capricious or erratic, it is likely to stand up to individual challenge and legal scrutiny in a court of law.

### Item difficulty or p-value

After administering a test, psychometricians analyse the effectiveness of each question using item analysis. The analysis of student response to assessment questions is a powerful tool for test improvement and remedial work. The Standards state, "the proportion answered correctly on the test may then be interpreted as an estimate of the proportion of items in the domain that could be answered correctly."^1^ This pertains to item difficulty index, which refers to the percentage of students who answered the question correctly on a particular test.

In the language of psychometrics, the item difficulty index is also called the p-value and should not be confused with the p-value related to statistical hypothesis testing. For example, if you have 60 students and 40 students get a question right, the p-value is for that question is 0.66 (40/60 = 0.66). The range of a p-value is between 0 and 1. High p-values, therefore, tell us the questions are easy (most students get them correct), and low p-values tell us the questions are difficult (most students get them wrong). Ordinarily, p-values between 0.25 and 0.75 represent a good item difficulty index.

If all students get a question correct or incorrect, then it does not discriminate between student ability and is not a good question and therefore needs review. The question is either too easy or too difficult for the cohort. Questions that are too easy or too difficult contribute little knowledge regarding students' ability and lead to low variability among marks. Low variance of marks reduces Cronbach's alpha (which is based on variance) and thus reduces the test-score reliability. In addition, if the vast majority of students get the question correct, it is hard to say whether the students have understood the material or whether the question was simply a bonus (too easy). Conversely, if the vast majority of students get the question incorrect, it is hard to interpret whether the students have not grasped the material or whether the question was simply beyond the students' ability. It should be noted that the p-value of a question does not reflect the item's property or difficulty in isolation but is a function of the interaction between the item and the student, where it bears a good relation to student ability within a specific test.

So, when reporting p-values, rather than stating the p-value of a particular question is 63%, it would be more correct, as Ebel and Frisbie suggest, to state "when this test was administrated to that particular group, its index of difficulty was 63%".^17^ This suggests that we should not expect similar p-values for assessments where students are different. If, suppose, there is a significant difference between p-values between previous and current students, we need to look at these two cohorts to determine if this difference is related to student ability or possibly a change related to the learning environment.

As noted previously, a question with a p-value of 100% means that everyone gets the question correct, and the question does not discriminate between high and low achieving students. This is a matter of discrimination index and is discussed below.

### Item discrimination index

Another index of item analysis is the Item Discrimination Index. Item discrimination refers to the capacity of a question to discriminate between high and low performers and helps judge the quality of a question. Practically, it is determined by calculating the difference between the percentage of high performers who answered a question correctly and the percentage of low performers who responded to a question correctly. If a question is answered correctly by students who score high marks overall and answered incorrectly by students who score low marks overall, the question is said to differentiate between 'those who know' and 'those who do not know' the material being tested. Questions with high discrimination indices increase test-score reliability. Therefore, designing challenging items within a moderate difficulty range increases the reliability of test scores. Very high discrimination indices, however, suggest a degree of question redundancy, and echo the information provided by other questions in the test.^18^

Psychometricians use various methods to calculate item discrimination indices. Item discrimination has a range between -1 to +1. A zero-item discrimination index indicates the question is not capable of discriminating high and low performers. Questions with a discrimination index greater than 0.20 are usually good questions. Questions with a very high discrimination index of >0.6/0.7 are unhelpful, as noted above and suggest redundancy.

Any question that has a negative item discrimination index must be carefully reviewed. This is not a good sign for the question as it indicates that more low performers answered the question correctly than high performers! This may indicate 1) ambiguity in the stem or that students are confused by the options as it is a tricky item, particularly for high performers, 2) all students answered the question by guessing as the question was very confusing, 3) the question measures something else compared to the rest of the assessment questions or most commonly 4)  the question has been mis-keyed (i.e., the wrong option has been chosen as the correct response when the question was designed. Any question with a negative item discrimination index needs to be reviewed carefully before the results are released to students, as such questions greatly contribute to systematic error in student marks.

We expect to see correct option has a positive item discrimination index. If this is not the case, we need to consider the points that have been raised above. Psychometricians also may use trace lines to show the plausibility of options in MCQs. Using trace lines can be useful for physicians to improve their assessment questions and, therefore, their exam cycles. An explanation of trace lines and examples of how they can be used is outside of the scope of this review but is discussed in an article for those interested in exploring it further.^4^

Aforementioned measures of reliability e.g., Cronbach’s alpha are based on overall test scores, and it is not possible to calculate such reliability scores for individual items. In order to select the most appropriate items for any test, it is therefore necessary to also consider both item reliability index and item validity index to obtain the best overall test score reliability. When using biserial point correlation to calculate the item discrimination index, multiplying this by the item standard deviation, produces an item reliability index, which as noted above indicates the internal consistency of the test at an individual item level. For reference, the sum of the item reliability indexes is also equal to the standard deviation of the total test score. The higher the test-score reliability, the higher the individual item reliability.

Multiplying the item discrimination index (obtained from an external criterion test total score) and the standard deviation of the item in question, an item validity index is produced, indicating the degree to which an item measures what is set out to measure.

Consider we have two tests, Test 1, and Test 2.  Assuming these tests measure the similar construct, and one test has predictive validity for the other, we can use this test as an external criterion to check validity of specific items in the other test. Say we want to select items to maximise score validity in Test 2. We use Test 1 as an external criterion for Test 2. We fist calculate the standard deviation of the item we wish to consider validity for in Test 2. We then calculate the item discrimination index using the total test scores for Test 1. If we multiply the item discrimination index calculated from Test 1 by the standard deviation of the item under consideration in Test 2 we will get the item validity index for this item in Test 2.

Therefore, to improve the test-score reliability and validity for the final test, select high item reliability and validity indices to provide reliable and valid information about the construct being measured. If the item reliability and validity indexes were not reported in the psychometric report, you might ask your psychometrician to provide them for you, as the Standards require assessment providers to show evidence of the process of individual item screening, noting that measures "such as item difficulty, item discrimination… should be documented".^1^

## Conclusions

The purpose of this article is to provide a simple guide for physicians as assessment developers and providers, to outline the basic psychometric measures that can be provided after administering any assessment and is structured to align with the Standards for educational and psychological testing^1^ which we encourage all those involved in this area of work to read and refer to. Psychometricians may provide other statistics in their reports that are not addressed in this article, however the references provided for this article may help clarify these further for those that are interested. We recommend that you consult your psychometricians to get a deeper understanding of the specific statistics they have provided if you are unfamiliar with them.

In conclusion, test-score reliability is the DNA of assessments, and assessment providers should report it for each assessment as evidence of such. Many factors can affect the reliability of test scores, e.g., quality of assessment questions, p-values, item discrimination index, the relationship between individual item responses and the total assessment mark, the spread of scores and test length. Physicians as medical educators can no longer be oblivious to the reliability and validity of the assessments they administer. This article hopefully goes some way to address their unmet learning needs.

### Conflict of Interest

The authors declare that they have no conflict of interest.
